# A mitochondria-targeted nano-platform for pancreatic cancer therapy

**DOI:** 10.3389/fchem.2022.951434

**Published:** 2022-09-21

**Authors:** Xiaoke Tan, Xin Zhu, Duanjie Xu, Yanmei Shi, Zhenzhen Wang, Mingzhuo Cao, Kai Hu, Lingzhou Zhao, Junwei Zhao, Mingsan Miao, Huahui Zeng, Xiangxiang Wu

**Affiliations:** ^1^ Academy of Chinese Medicine Sciences, Henan University of Chinese Medicine, Zhengzhou, China; ^2^ Pharmacy College, Henan University of Chinese Medicine, Zhengzhou, China; ^3^ Department of Nuclear Medicine, Shanghai General Hospital, Shanghai Jiao Tong University School of Medicine, Shanghai, China; ^4^ Department of Clinical Laboratory, The First Affiliated Hospital of Zhengzhou University, Zhengzhou, Henan, China

**Keywords:** triptolide, stachydrine, liposome, pancreatic cancer, mitochondria, apoptosis

## Abstract

Liposome is a conventional drug delivery system which has been widely used in the pharmacy field. However, its applications are greatly restricted in clinical practice by the disadvantages of cholesterol and nonselective distribution. Herein, a novel platform for anti-tumor drug delivery was developed by incorporating an amphiphilic stachydrine-octadecane conjugate (SS) as the mitochondria-targeting molecule onto the triptolide-liposome surfaces (SS-TP LPs). The polyethylene glycol (PEG) and the suitable particle size (about 133 nm) of liposomes facilitated their stabilities, the long half-life in blood and the escape from the rapid elimination. The SS-TP LPs were internalized and accumulated into the mitochondria of cancer cells in a time-dependent manner, followed by triggering permeabilization of the mitochondrial outer membrane by inhibiting Bcl-2, and then further caused greater cancer cell death *via* releasing cytochrome C and initiating a cascade of caspase 3 reactions. In the Pan02 tumor-bearing mice, the SS-TP LPs showed significant efficacy in inhibiting tumor growth and reducing tumor size but synchronously exhibited specific mitochondria-targeting and much lower subacute toxicity compared with the free TP and TP LPs. Our study suggests that SS-TP LPs can be a promising anticancer drug delivery system for mitochondria-targeted therapy in pancreatic cancer.

## 1 Introduction

Pancreatic cancer is among the most lethal malignancies worldwide, with a 5-years survival rate of about 5% (McGuigan et al., 2018). Many chemotherapeutic drugs, such as gemcitabine, 5-fluorouracil (5-FU), and albumin-paclitaxel nanoparticle, exhibit potent activity against pancreatic cancer in animal models but demonstrate unsatisfied outcomes in the clinic (Giri et al., 2017; Chugh et al., 2012; Wang et al., 2016). Furthermore, gemcitabine is still the first-line chemotherapeutic drug for pancreatic cancer so far, despite showing a median survival of only 6 months and serious adverse effects among patients. Recently, although several advanced nanoengineering approaches for target-specific delivery of drug cocktails, such as gemcitabine and cisplatin, gemcitabine and paclitaxel, gemcitabine and OMe-PS-miR-519c, have enhanced the therapeutic efficacy in animal models, they still face major challenges due to the proper ratiometric delivery of multiple drugs and the extra toxic side effects from the cisplatin, paclitaxel, and so on (Xin et al., 2020; Shabana et al., 2021; Tarannum et al., 2022). Hence, effective and safe chemotherapeutics and therapeutic strategies are still urgent needs to improve the treatment of pancreatic cancer.

Many anti-cancer drugs can induce the suicide of cancer cells by apoptosis or kill cancer cells by their cytotoxicity. Apoptosis, namely, programmed cell death, is a normal process involved in cellular stress response. The drug-induced apoptosis includes two main pathways, namely, “triggering the cellular stress to release apoptogenic factors” and “activating upstream caspases”, which are initiated at the site of mitochondria (Green, 1998). Hence, the mitochondrion has been recently considered a potential drug delivery target in cancer therapy. Mitochondria play critical roles in the conversion of energy, cellular metabolism, signal hub, cell proliferation, programmed cell death, and protein synthesis in normal as well as cancer cells (Nunnari and Suomalainen, 2012). Thus, selective mitochondrial targeting in the cancer cells instead of the normal cells is the key to cancer therapeutics. Due to the highly negatively charged mitochondria of the cancer cells, several mitochondrion-targeted nanovectors were developed to deliver drugs successfully into tumor tissues through various cationic moieties mediated targeting (including triphenylphosphine, peptide, and guanidinium) (Faria et al., 2021; Jiang et al., 2021; Ye et al., 2021). The combination of the cationic molecule and nanotechnology was a promising strategy for cancer therapy to improve drug uptake, reduce drug ejection, and maximize therapeutic efficacy (Wang et al., 2020; Qi et al., 2021).

In recent years, triphenylphosphine with a delocalized positive charge has been used as a classically mitochondrion-targeted moiety on the surface of nanoparticles, which makes it easy to pass through the mitochondrial membrane and accumulate in the mitochondria. For instance, triphenylphosphine-based polylactic acid nanoparticles (TPP-PLLA NPs) were developed to improve the mitochondrion-targeted delivery efficiency of anticancer drugs (Ye et al., 2021). In addition, a series of reported peptide-based nanoparticles (such as MTS-peptides/pND1 nanoparticles) showed suitable properties concerning the physicochemical properties and mitochondrial targeting ability, which could be applied in gene therapy or chemotherapy for cancer (Faria et al., 2021). Meanwhile, Jiang et al. reported a guanidinium-based dendritic lipopeptide (DLP) liposome which exhibited higher mitochondrion-targeting delivery efficiency than the triphenylphosphine-based nano-system (Jiang et al., 2021). Although DLP liposome was easy to overcome multiple biological barriers for sub-cellular delivery upon the mitochondrion accumulation and tumor eradication, the guanidinium-based dendritic lipopeptides moiety had the disadvantages of complex chemical structure, large molecular weight, and tedious chemical synthesis. As a consequence, it is essential to develop simple and obtainable mitochondrion-targeting nanoparticles for the improvement of therapeutic efficacy in cancer patients.

To address these issues, stachydrine (N, N-Dimethyl-L-proline), as the mitochondrion-targeted moiety, a natural small molecule, was first introduced onto the surface of nanoparticles for mitochondrion-targeting delivery. Stachydrine is a positively charged ingredient from the leaves of motherwort, which has various bioactivities, including anti-cancer, anti-inflammatory, and decreasing oxidative stress (Cheng et al., 2020). Stachydrine exerts anti-cancer effects mainly through inducing apoptosis and inhibition of cell proliferation (Liu et al., 2018; Zhao, 2018). Triptolide (TP), extracted from the Tripterygium wilfordii Hook F, is superiorly effective against pancreatic cancer in comparison with gemcitabine and paclitaxel *in vitro*/vivo (Wang et al., 2016) (Herreros-Villanueva et al., 2012; Zhu et al., 2012; Li et al., 2016). Triptolide can induce cell death in pancreatic cancer by multiple pathways, such as apoptosis and autophagy (Phillips et al., 2007; Mujumdar et al., 2010). However, its potential clinical use is restricted to poor solubility in water and toxicity (Shamon et al., 1997; Green, 1998). Liposomes are able to improve the therapeutic efficacy of drugs and decrease the systemic toxicity. To obtain a long blood circulation and cancer targeting of triptolide, polyethylene glycol (DPPE-mPEG2000) and stachydrine derivative (1,1-dimethylpyrrole-2-octracyl formate-1-ammonium chloride, SS) have been used in the formulation. Hence, the drug-loaded liposomes (SS-TP LPs) containing lecithin (LC), DPPE-mPEG2000, triptolide, and SS can prolong the half-life of drug in the blood and increase the cellular uptake of the drugs.

In the present study, the mitochondrial targeting molecule (SS) was synthesized using the one-step method and introduced onto the TP-liposome surface. We hypothesized that the SS-TP LPs had the mitochondria-targeting property, low toxicity, and could induce cancer cell apoptosis by mediating mitochondrial damage. Therefore, the objectives of our study were the development of the mitochondria-targeting nanoparticles, the exploration of the action mechanisms, and the evaluation of the anti-cancer efficacy in mice.

## 2 Materials and methods

### 2.1 Materials

Stachydrine was purchased from Ci Yuan Biotechnology Co., Ltd., (Shanxi, China). Triptolide was purchased from Xi’an Haoxuan Biotechnology Co., Ltd., (Shanxi, China). DPPE-mPEG_2000_ was obtained from Sigma-Aldrich (St. Louis, MO, United States). Cell Counting Kit-8 (CCK-8) was obtained from Abmole. 1-Octadecanol. Lecithin and coumarin 6 were purchased from Shanghai Aladdin Biochemical Technology Co., Ltd., (Shanghai, China). Mito-Tracker Red CMXRos, Lyso-Tracker Red, 1,1'-dioctadecyl-3,3,3',3'-tetramethylindodicarbocyanine,4-chlorobenzenesulfonate salt (DID) were provided by Beyotime (Shanghai, China). Annexin V-FITC/PI apoptosis kit was purchased from Multi Sciences (China). 4',6-diamidino-2-phenylindole (DAPI), reactive oxygen species assay kit, and 5.5’,6.6’-tetrachloro-1.1’,3.3’-tetraethylbenzimidazolylcarbocyanine iodide (JC-1) were purchased from Solarbio. Anti-caspase-3 antibody, anti-Bcl-2 antibody, and anti-Bax antibody were purchased from Proteintech. Anti-cytochrome C (Cyt C) antibody was purchased from Cell Signaling. Anti-β actin antibody was purchased from Santa Cruz. All other reagents were purchased from commercial suppliers and were of analytical grade.

The murine-derived pancreatic cancer cells (Pan02) were supplied by BeNa Culture Collection (China). C57BL/6J mice (female, 6–8 weeks) were purchased from Jinan Pengyue Experimental Animal Breeding Co., Ltd., which were maintained under standard housing conditions. All animal experimental protocols were performed in accordance with institutional guidelines and approved by the Experimental Animal Ethics Committee of Henan University of Chinese Medicine (Approval ID: DWLL201903010).

### 2.2 Synthesis of (2S)-1,1-dimethylpyrrole-2-octracyl formate-1- ammonium chloride

Stachydrine (2.07 g, 14.4 mmol) was added to SOCl_2_ (4 ml) and stirred for 1.5 h at room temperature. After removal of the residual SOCl_2_ under vacuum, the residues were dissolved in anhydrous CH_2_Cl_2_ (10 ml) contained anhydrous potassium carbonate (2 g, 14.4 mmol), followed by the addition of 1-octadecanol (3.89 g, 14.4 mmol), and were stirred for 3 h under the ice bath. The reaction was ended with water, and the residues were separated and purified with silica gel column (CH_2_Cl_2_/MeOH = 30:1) to obtain pale yellow solid [(2S)-1,1-dimethylpyrrole-2-octracyl formate-1-ammonium chloride (SS), yield 68.9%]. The stachydrine-octadecane conjugate was characterized by NMR (500 MHz, AV500 + BH0055, Bruker) and HRMS (TSQ ALTIS, Thermo Fisher Scientific Inc.). ^1^H NMR (500 MHz, Chloroform-*d*) δ 5.36 (t, *J* = 9.7 Hz, 1H), 4.54 (q, *J* = 10.3 Hz, ^1^H), 4.27–4.11 (m, ^2^H), 4.04 (dd, *J* = 11.6, 8.0 Hz, ^1^H), 3.75 (s, ^3^H), 3.22 (s, ^3^H), 2.76 (dd, *J* = 13.7 Hz, 9.7 Hz, 4.2 Hz, ^1^H), 2.42 (d, *J* = 16.8 Hz, ^1^H), 2.30–2.06 (m, ^2^H), 1.64 (*p*, *J* = 6.9 Hz, ^2^H), 1.24 (s, ^30^H), 0.86 (t, *J* = 6.7 Hz, ^3^H). MS (ESI) m/z, Calcd for C_25_H_50_NO_2_ (M + H) ^+^397.39, found 397.63 (M + H)^+^.

### 2.3 Preparation of SS-TP LPs

Lecithin (LC), SS, DPPE-mPEG2000, and TP were dissolved in methanol (MeOH) with a weight ratio of 10:1:0.4:0.4. Methanol was removed under vacuum to form lipid film and dried thoroughly for 12 h. The film was hydrated using 4 ml of water for 2 h at 50°C, followed by ultrasonic dispersion and extrusion through a polycarbonate membrane (pore size: 100 nm). Self-assembled nanoparticles formed were centrifugated with ultrafilter (Millipore, Amicon Ultra, cut off: 30 kDa) at 4,000 r/min for 30 min at 4°C. Finally, targeting triptolide liposomes (SS-TP LPs) were produced.

Accordingly, the triptolide liposomes (TP LPs), blank liposomes, and blank targeting liposomes (SS LPs) were prepared using the same procedures as mentioned earlier. In addition, the coumarin liposomes (C6 LPs), targeting coumarin liposomes (SS-C6 LPs), DID liposomes (DID LPs), and targeting DID liposomes (SS-DID LPs) were also prepared as the fluorescent probes, of which the coumarin 6 (C6) and DID were used instead of triptolide in these liposomes, respectively.

### 2.4 Characterization of SS-TP LPs

#### 2.4.1 Particle size and zeta potential determination

The size, polydispersity index, and zeta potential of the prepared nanoparticles were monitored using a dynamic light scattering analyzer (DLS; NanoBrook 90 plus PALS, Brookhaven, United States) (Qiao et al., 2020).

#### 2.4.2 Transmission electron microscopy image analysis

The prepared nanoparticle suspensions (100 µL) were stained negatively with 2% (w/v) of phosphotungstic acid solution, placed on a copper grid coated with carbon film, and then air-dried at room temperature. The morphologies of the nanoparticles were observed by transmission electron microscopy (TEM; JEM-1400, JEOL Ltd., Tokyo, Japan).

#### 2.4.3 Encapsulation efficiency and loading capacity

The encapsulation efficiency (EE, %) and loading capacity (LC, %) of the liposomes were measured using the high-performance liquid chromatography (HPLC) method. The prepared liposomes were dialyzed to remove the un-encapsulated TP, C6, or DID. After dialysis and lyophilization, a given amount of liposome was destroyed by methanol. The HPLC (Agilent 1,260, United States) (Xiong et al., 2019) with a UV detector at 218 nm and a reverse phase C18 column eluted with acetonitrile–ddH_2_O (33:67 vol%) at 1.0 ml/min flow rate, followed by calculation according to the following formula:

$$EE\left(\%\right)=\left(M_{i}/M_{total}\right)\times 100\%;LC\left(\%\right)=\left(M_{i}/M_{Lip}\right)\times 100\%,$$ (1)

where M_i_ is the weight of TP, SS, C6, or DID in the liposomes; M_total_ is the total weight of the encapsulated and un-encapsulated TP, SS, C6 or DID in the liposome suspensions; and M_Lip_ is the weight of the liposomes after lyophilization.

#### 2.4.4 *In vitro* stability

To evaluate the *in vitro* stability of SS-TP LPs, the test liposomes were incubated in water, PBS (pH 7.4), mouse serum, and DMEM medium (10% FBS) at 4 or 37°C, respectively. The particle size, polydispersity index, and zeta potential of the test liposomes were monitored at predetermined time intervals (12, 24, 36, and 48 h; or 3, 6, 9, 12, and 15 days).

#### 2.4.5 Release kinetics

The release rate (RR, %) of TP from SS-TP LPs was measured using the dialysis method in the release medium [PBS (pH 7.4) containing Tween 80 (0.5%, v/v)]. About 1 ml of TP, TP LPs, or SS-TP LPs (containing 0.50 mg of TP) suspensions was sealed in a dialysis bag (interception, 2,000Da). The bag was immersed into 30 ml of release medium at 37°C with persistent and mild stirring at a speed of 60 rpm. A volume of 0.5 ml dialysate was taken and synchronously restored with an isometric fresh release medium at a given time point. The content of TP in the dialysate was measured using HPLC as earlier. Each assay was repeated in triplicate. The release rate was calculated according to the following formula:

$$RR\left(\%\right)=\left(M_{i}/M_{total}\right)\times 100\%,$$ (2)

where M_i_ is the weight of TP in dialysate and M_total_ is the weight of TP in the liposome before the release experiment.

### 2.5 Cell viability assay

The pancreatic cancer cells at 5 × 10^3^ per well were plated in 96-well plates for 24 h under the condition of 5% CO_2_ at 37°C. Cells were preincubated with the blank targeting liposomes (SS LPs), TP (5 nmol/ml, 10 nmol/ml, 20 nmol/ml, 40 nmol/ml, 80 nmol/ml, 160 nmol/ml, 320 nmol/ml, and 640 nmol/ml in DMEM with 1‰ DMSO), TP LPs, or SS-TP LPs (at equivalent concentration of TP) for 24 h. Blank wells were added with culture medium as the blank control, and cells were added with culture medium as the control. The cell viability was measured by CCK-8 assay. The optical density of the solution was detected by a microplate reader (Thermo, United States) under 450 nm absorbance values. Each assay was repeated in triplicate. The survival rates were calculated according to the following formula:

$$Cell\;viability\left(\%\right)=\left(\left(A_{sample}-A_{blank}\right)/\left(A_{control}-A_{blank}\right)\right)\times 100\%,$$ (3)

where A_sample_, A_control_, and A_blank_ are the absorbance value of the sample, the control, and the blank control.

### 2.6 Analysis of apoptosis

Pan02 cells at 2 × 10^5^ per well in a 6-well plate were preincubated for 24 h under the condition of 5% CO_2_ at 37°C. After 24-h incubation with culture medium, TP (60 nmol/L), TP LPs, or SS-TP LPs (containing 60 nmol/L TP), cells were centrifuged (1,000 r/min, 3 min) and washed with PBS (twice), and then incubated in 500 μL of binding buffer with 10 μL of PI and 5 μL of Annexin V-FITC for 5 min. The apoptosis rate in various groups was measured by flow cytometry (CytoFLEX, Beckman Coulter, United States).

### 2.7 ROS level analysis

Pan02 cells were seeded at 5 × 10^4^ per well in CLSM plates and cultured at 37°C for 24 h. After the incubation with culture medium, TP (60 nmol/L), TP LPs, or SS-TP LPs (containing 60 nmol/L TP) for 6 h at 37°C, the culture media were removed, and cells were incubated with DCFH-DA serum-free media (500 μl, 10 μmol/L) according to the manufacturer’s protocol for 20 min. ROS level was determined by confocal laser scanning microscopy (CLSM) at 525 nm fluorescence emission wavelength.

### 2.8 Western blot analyses

Pan02 cells at 2 × 10^5^ per well in a 6-well plate were preincubated for 24 h under the condition of 5% CO_2_ at 37°C. After 24-h incubation with culture medium, TP (60 nmol/L), TP LPs, or SS-TP LPs (containing 60 nmol/L TP), the cells were lysed by RIPA buffer and centrifuged at 12,000 rpm for 10 min at 4°C. The supernatants were collected for assessment of the total proteins by Western blot analysis. The total protein levels were quantified by BCA protein assay using a microplate reader following the manufacturer’s instructions (NCM Biotech, China). Samples were subjected to SDS-PAGE with a 10% acrylamide gel and transferred to PVDF membrane. Membranes were blocked with 5% non-fat milk for 1.5 h at room temperature and then incubated at 4°C overnight with the following primary antibodies: anti-cytochrome C, anti-caspase-3, anti-Bcl-2, and anti-Bax with a 1:1,000 dilution. Subsequently, membranes were incubated with the horseradish peroxidase (HRP)-conjugated goat anti-rabbit (goat anti-mouse) secondary antibody (1:5,000 dilution) for 1.5 h at room temperature. The bands were visualized using a chemiluminescent HRP substrate detection kit. The gray value of protein bands was quantitated by determination of the intensity of the hybridization signals *via* the Alpha software.

### 2.9 Cellular uptake

Pan02 cells at 5 × 10^4^ per well in a 6-well plate were preincubated for 24 h under the condition of 5% CO_2_ at 37°C. Subsequently, the cells were treated with C6 LPs and SS-C6 LPs for 1 and 3 h, respectively. The final concentration of coumarin 6 was 200 μg/ml. After incubation, the cold PBS was used to stop the cellular uptake, and the cells were washed thrice with PBS. The fluorescence intensity in various groups was measured by flow cytometry.

### 2.10 Lysosome localization and permeabilization

About 5 × 10^4^ Pan02 cells per well were seeded into a 24-well plate with glass coverslips. After 12-h incubation, the cells were treated with C6 LPs and SS-C6 LPs for 1, 2, and 4 h, respectively. After PBS wash, the cells were incubated with Lyso-TrackerTM Red (2.0 ml, 200 nmol/L) in the dark for 20 min, followed by incubation with DAPI (50 μl, 10 μg/ml) for 10 min. Cells were washed thrice with PBS and then fixed with 4% tissue cell fixation solution (Solarbio) at 37°C for 10 min. The coverslips were imaged by CLSM.

Pearson’s correlation coefficient (PCC): The scatterplots can be represented in quantifying fluorescence colocalization using Pearson’s correlation coefficient as a statistic. The PCC formula is shown below with an image consisting of red and green channels.

$$PCC=\frac{\sum_{i}\left(R_{i}-\bar{R}\right)\times \left(G_{i}-\bar{G}\right)}{\sqrt{\sum_{i}{\left(R_{i}-\bar{R}\right)}^{2}\times \sum_{i}{\left(G_{i}-\bar{G}\right)}^{2}}},$$ (4)

where *R*_*i*_ (‾*R*) and *G*_*i*_ (‾*G*) refer to the (mean) intensities of the red and green channels across the entire image, respectively. PCC value at a range of 1 shows that fluorescence intensities are linearly related between two images; inversely, PCC of -1 represents a negative correlation. PCC value near zero reflects distributions of probes that are uncorrelated with one another.

### 2.11 Mitochondria localization

About 5 × 10^4^ Pan02 cells per well were seeded into a 24-well plate with glass coverslips. After 12-h incubation, the cells were treated with C6 LPs and SS-C6 LPs for 1, 3, and 6 h, respectively. The cells were washed twice with PBS. Mitochondria were labeled with Mito Tracker TM Red (2.0 ml, 200 nmol/L) in the dark for 20 min, and cell nucleus were labeled with DAPI (50 μl, 10 μg/ml) for 10 min. Cells were washed thrice with PBS and then fixed with 4% tissue cell fixation solution for 10 min at 37°C. After PBS wash, the coverslips were subjected to fluorescence imaging by CLSM.

### 2.12 JC-1 assay

A total of 5 × 10^4^ Pan02 cells per well were seeded in CLSM plates and cultured for 24 h at 37°C. After incubation with the culture medium, TP, TP LPs, and SS-TP LPs (containing 60 nmol/ml TP) for another 24 h, cells were washed thrice with PBS and then incubated with a cationic dye, JC-1 dye (1.0 ml, 10 μg/ml), in the dark for 20 min at 37°C. The JC-1 solution was discarded and washed twice with buffer solution. The JC-1 dyes in cells were observed by CLSM at 529 and 590 nm fluorescence emission wavelength.

### 2.13 Bio-distribution of SS-TP LPs

C57BL/6J mice (14 g ± 2 g) were subcutaneously injected with 1 × 10^6^ Pan02 cells (0.1 ml) on the right flanks. When nodules with volume of about 100 mm^3^ appeared in the inoculation region, the tumor-bearing mice were chosen as the successful models for the next experiments. The mice were given intravenous injection (iv) with DID, DID LPs, and SS-DID LPs (200 μL), respectively. After the mice were anaesthetized, the bio-distribution behaviors were visualized at given time points by using an IVIS^®^ Lumina Series 3 system (PerkinElmer, United States) equipped with fluorescent filter sets (excitation/emission, 648/670 nm). After the whole-body imaging, the anaesthetized mice were euthanized and dissected, washed with PBS, and the lung, liver, kidney, spleen, heart, and tumor were weighed. The *ex vivo* fluorescence imaging was also carried out using the same settings, followed by the fluorescent quantitation analysis using the Living Image-4.5 software according to standard protocols.

### 2.14 *In vivo* antitumor effect

When the volume of tumor nodules reached about 80 mm^3^, the mice (*n* = 8) were injected intravenously with saline, TP, TP LPs, or SS-TP LPs (equivalent to TP 0.4 mg/kg) and were weighed synchronously every two days. At the same time, tumor volume (mm^3^) was measured using calipers and calculated by the equation (Legends × Width^2^/2). On day 14, blood samples were collected from the eyes, and the mice were then sacrificed in accordance with institutional guidelines. The tumors were dissected, washed with PBS, and weighed.

### 2.15 Histological assay

The lung, liver, kidney, spleen, heart, and tumor tissues were embedded in paraffin after formalin was fixed and sectioned at 5 μm thickness for further analysis. The sections were stained with H&E and visualized by fluorescence microscopy according to the operation manual. The apoptotic tumor cells were also evaluated using the TUNEL Apoptosis Assay Kit (Servicebio, China) according to the recommended instructions and examined by fluorescence microscopy (Axiscope5, Carl Zeiss, Germany). The immunohistochemistry (IHC) staining of Ki67 was examined using a Ki67-antibody Assay Kit (Servicebio, China) according to standard protocols.

### 2.16 *In vivo* safety

The tumor-bearing mice were weighed and injected intravenously with saline, TP, TP LPs, or SS-TP LPs (equivalent to TP 0.4 mg/kg) every two days. On day 14, blood samples were collected from the eyes, and the mice were then sacrificed in accordance with institutional guidelines. The lung, liver, kidney, spleen, and heart were dissected and washed with PBS. All of the serum samples were used to analyze the liver/kidney function (Nanjing Jiancheng Bioengineering Institute, China).

### 2.17 Statistical analysis

All data were analyzed using SPSS 17.0 statistical package (SPSS, Inc., Chicago, IL, United States). The data are expressed as the mean ± SD from at least three independent experiments. Statistical analysis was performed using the two-tailed Student’s *t*-test. *p < 0.05, **p < 0.01, and ***p < 0.001 were considered statistically significant.

## 3 Results and discussion

### 3.1 Synthesis and characterization of SS-TP LPs

**FIGURE 1:**
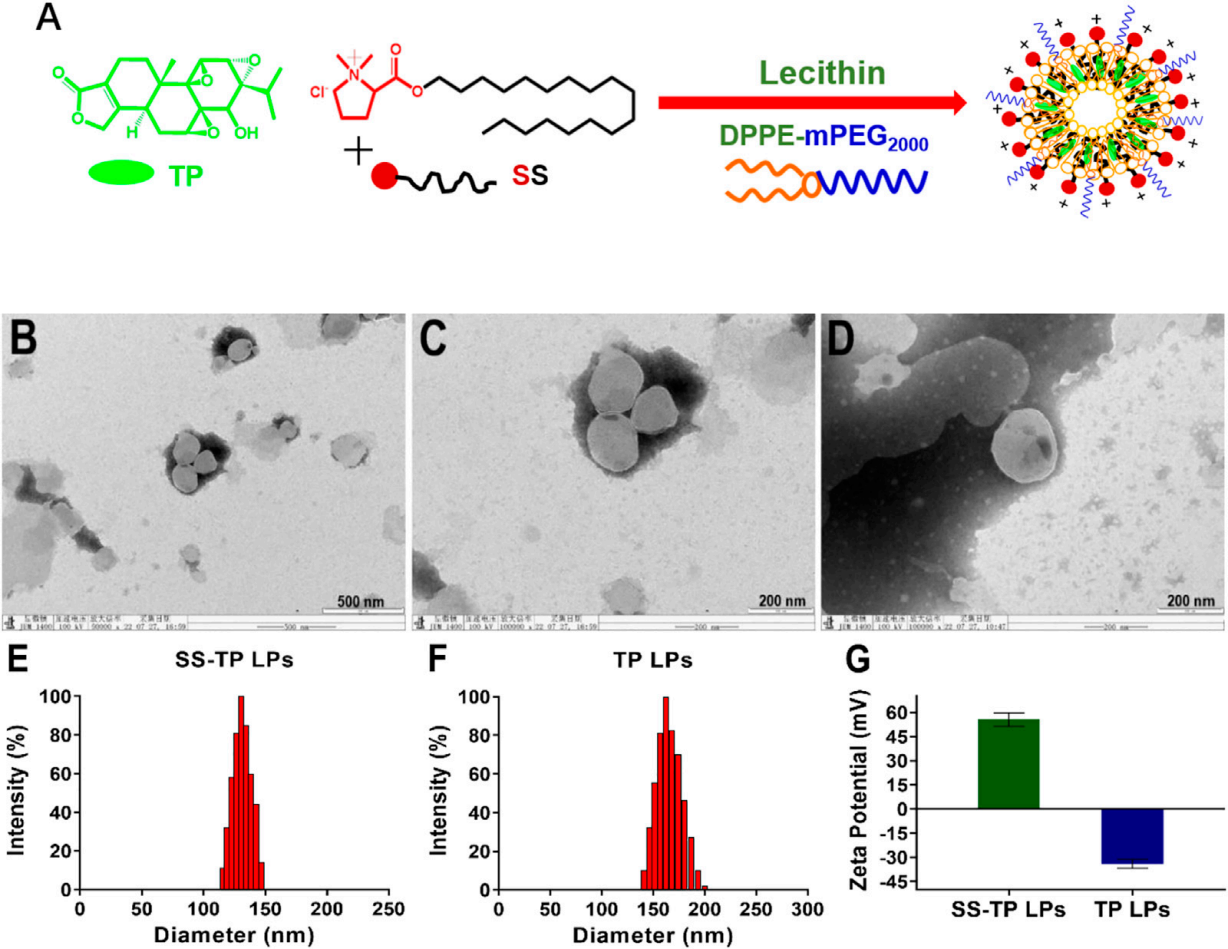
Schematic illustration of preparation, morphological identification, particle size distributions, and Zeta potential determination of SS-TP LPs. Preparation scheme of SS-TP LPs. **(A)** TEM images of SS-TP LPs **(B and C)** and TP LPs **(D)** determined by transmission electron microscope; particle size distribution **(E and F)** and zeta potential **(G)** of SS-TP LPs and TP LPs determined by DLS, respectively (*n* = 3).

A mitochondrion targeting head, (2S)-1,1-dimethylpyrrole-2-octracyl formate-1-ammonium chloride (SS, yield 68.9%), was first synthesized *via* the esterification reaction using thionyl chloride between stachydrine and 1-octadecanol (Figure 1A). The structure of obtained SS was characterized by 1H-nuclear magnetic resonance spectroscopy (^1^H-NMR) and high-resolution mass spectroscopy (HRMS). The chemical shifts for the methylene (-COO-CH_2_-, 4.11 ppm–427 ppm) and the 2-position of stachydrine (N-CH(CO-)CH_2_-, 5.36 ppm) were exhibited in the 1H NMR spectra of the compound SS. The precise molecular weight detected by HRMS was uniquely associated with a specific molecular formula.

SS-TP LPs were prepared by mixing LC, SS, and TP in a weight ratio of 10:1:0.4. The DPPE-mPEG_2000_ was used to coat the surface of nanoparticles with polyethylene glycol (PEG) to evade the mononuclear phagocyte system (MPS) and prolong circulation in blood. The encapsulation efficiency (EE) and loading efficiency (LE) of triptolide were measured using the HPLC method, and the EE of TP in SS-TP LPs was 62.67 ± 4.59%, and the LE was 0.76 ± 0.01%. TEM showed clear outlines of SS-TP LPs with a diameter of about 133 nm **(** Figures 1B and C **)** and TP LPs with 166 nm **(** Figure 1D **)**. The mean hydrodynamic diameter and zeta potential of SS-TP LPs and TP LPs varied from 130 nm to 162 nm and PDI value from 0.132 ± 0.04 to 0.186 ± 0.01 and change from ^+^55.60 mV ± 4.12 mV to -34.01 mV ± 2.75 mV, respectively **(** Figures 1E–G **)**. The zeta potential changes confirmed the successful insertion of SS into the surface of nanoparticles. The size of SS-TP LPs showed an optimal diameter range of 100 nm–160 nm, which would ameliorate the half-life in blood circulation and accumulation in the tumor tissues by improving the unique leaky vasculature and retention effect (Wang et al., 2015).

**FIGURE 2:**
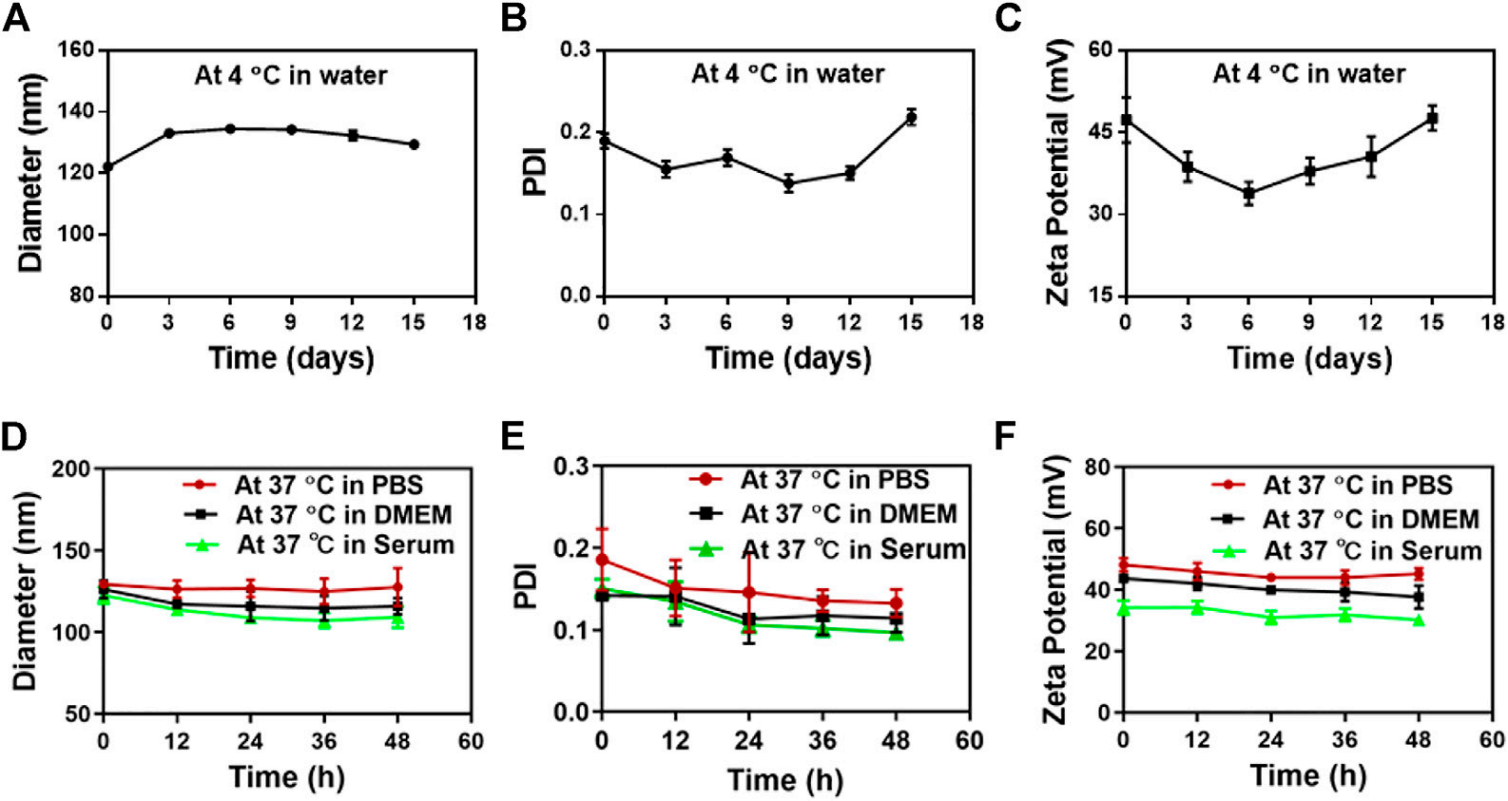
Stability of SS-TP LPs determined by DLS under varying conditions. **(A-C)** The particle size, PDI, and zeta potential of SS-TP LPs in water at 4°C under storage for 15 days (*n* = 3). **(D-F)** The particle size, PDI, and zeta potential of SS-TP LPs in PBS, mouse serum, and DMEM with 10% FBS at 37°C for 48 h, respectively (*n* = 3).

The *in vitro* stability of SS-TP LPs at 4°C and 37°C was investigated in water, PBS, DMEM with 10% FBS, or mouse serum. The strong potential of nanoparticles makes a repulsive force for each other to create dispersion stability. Therefore, the particle sizes and zeta potential changes are able to be used as a stability indicator of nanoparticles. The SS-TP LPs at 4°C remained intact in water even after 15 days because the particle sizes and zeta potential did not significantly change. The diameter of SS-TP LPs increased from 122 nm ± 1 nm to 129 nm ± 1 nm over 15 days (Figure 2A). The PDI value increased from 0.19 ± 0.01 to 0.22 ± 0.01 (Figure 2B). Meanwhile, the zeta potential also varied from 47.29 mV ± 4.15 mV to 47.61 mV ± 2.33 mV over 15 days (Figure 2C). The stability of SS-TP LPs at 37°C was detected within 48 h in PBS, DMEM with 10% FBS, and serum, respectively. Figures 2D–F show the slight decrease in the size, PDI, and zeta potential of SS-TP LPs dispersed in various media at 37°C. However, the parameter of the size, PDI, and zeta potential in serum decreased more than that in PBS and DMEM. As shown in Figures 2D–F, the diameter of SS-TP LPs slightly decreased from 129 nm to 117 nm, and the PDI value also varied from 0.22 ± 0.01 to 0.11 ± 0.01. At the same time, the zeta potential decreased from 48.21 mV ± 1.61 mV to 34.21 mV ± 4.64 mV. The results indicated that SS-TP LPs were stable even without cholesterol in storage condition for 15 days and in PBS, mouse serum, or DMEM with 10% FBS for 48 h.

**FIGURE 3:**
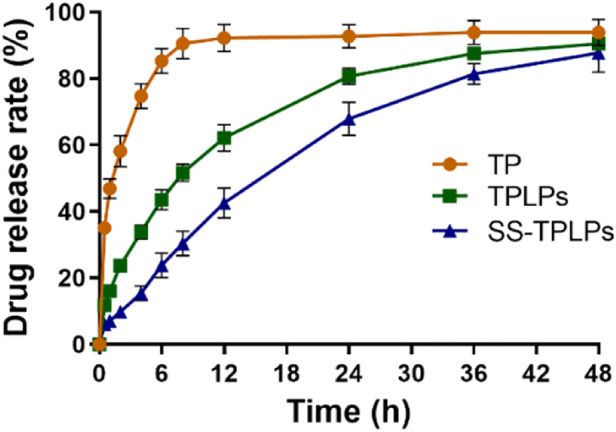
Controlled release manner of TP LPs and SS-TP LPs. Free TP, TP LPs, or SS-TP LPs dissolved in a dialysis bag and shaken at 37°C at 100 rpm (*n* = 3).

The *in vitro* drug release from nanoparticles described the controlled release ability of SS-TP LPs in the simulated body fluids. Figure 3 illustrates that the free TP was released in a burst-release manner out of the dialysis bag, whereas TP from SS-TP LPs and TP LPs was released in a sustainable manner. The release rate of free TP from the dialysis bag was ∼90.56% at the initial 8 h, followed by much slight release. Nevertheless, a similar release trend of TP from SS-TP LPs and TP LPs was observed within 48 h. 87.56% of TP was released from TP LPs at the initial 36 h, and approximately 90.47% until 48 h. Compared to TP LPs, SS-TP LPs showed a relatively slow and delayed release of TP, and the release rate reached 87.68% within 48 h. Compared to TP LPs, the positively charged SS was introduced onto the surface of SS-TP LPs, which increased the Zeta potential of nanoparticles from -34.01 mV ± 2.75 mV of TP LPs to ^+^55.60 mV ± 4.12 mV of SS-TP LPs. Nevertheless, the drug release rate of SS-TP LPs was contrary to their stabilities which might depend mainly on the Zeta potential, particle size and others of nanoparticles. The high absolute value of Zeta potential means more stable nanoparticles when other factors remain unchanged. Hence, the drug release rate of SS-TP LPs is lower than that of TP LPs. SS-TP LPs might maintain a sustainable and suitable blood drug concentration, which would enable great reduction of the drug dose and the usage frequency.

### 3.2 *In vitro* cytotoxicity, apoptosis, ROS production, and mechanism

**FIGURE 4:**
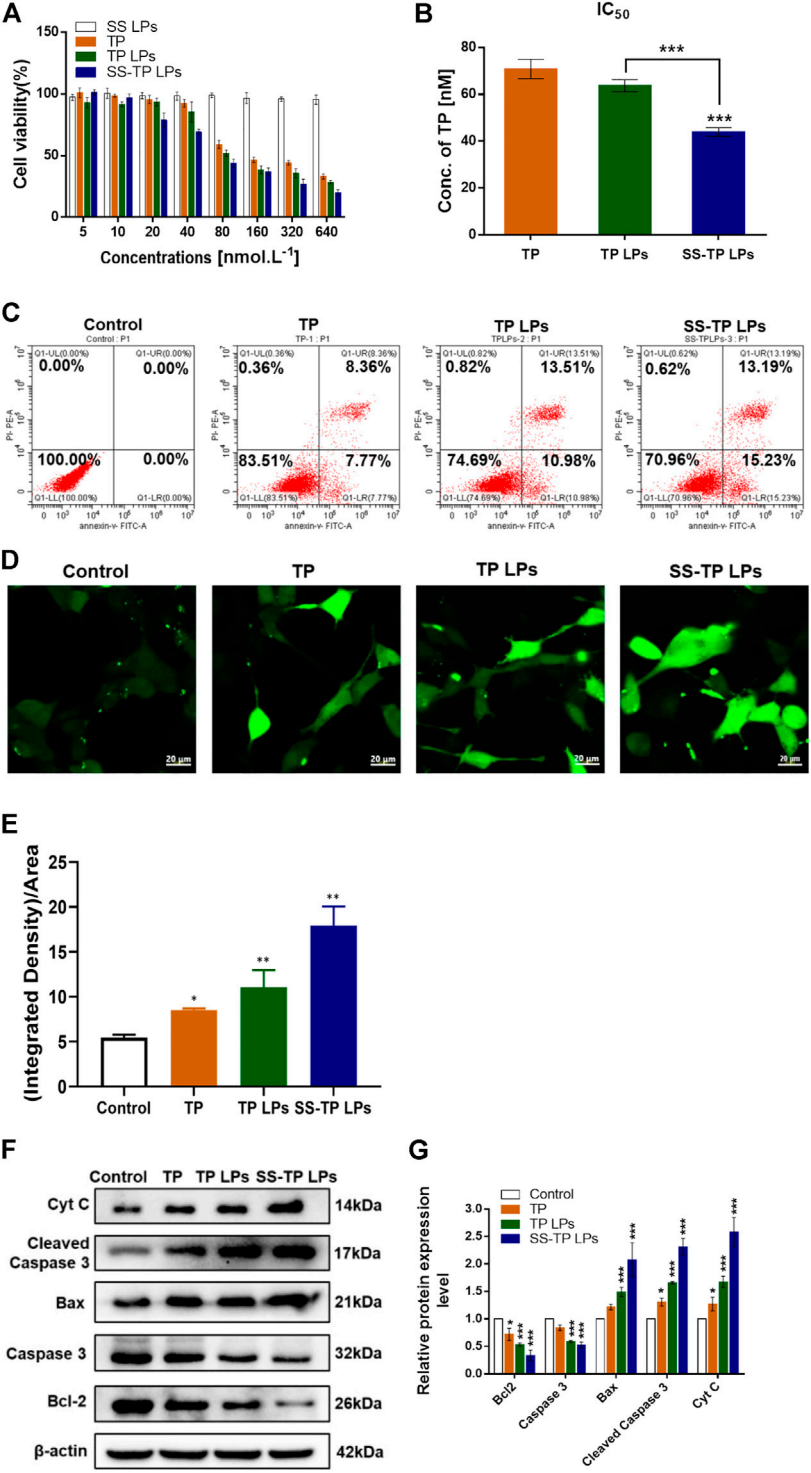
**(A)** Survival rates (%) of Pan02 cells after incubation with SS LPs (Blank targeting LPs), free TP, TP LPs, or SS-TP LPs at 24 h. **(B)** IC_50_ of Pan02 cells after incubation with TP, TP LPs, and SS-TP LPs. **(C)** Flow cytometric analysis of apoptosis induced by TP, TP LPs, and SS-TP LPs in Pan02 cells (lower left quadrant shows the percentage of healthy cells, lower right quadrant represents the percentage of early apoptotic cells, upper right quadrant shows the late apoptotic cells, and upper left quadrant represents the percentage of necrotic cells after treatment with TP, TP LPs, and SS-TP LPs for 24 h). **(D)** Fluorescent images of ROS generation in Pan02 cells determined by CLSM. scale bar = 20 μm. **(E)** Quantification analysis of the ROS generation. **(F)** Western blot analysis to visualize the expression of Bcl-2, Bax, cytochrome C, cleaved caspase 3, and pro-caspase 3 after treatment of Pan02 cells with TP, TP LPs, and SS-TP LPs for 24 h. **(G)** Quantification of Bcl-2, Bax, Cyt C, cleaved caspase 3, and pro-caspase 3 expressions in Pan02 cells after incubation with TP, TP LPs, and SS-TP LPs for 24 h from Western blot. *p < 0.05 and **p < 0.01, were considered statistically significant.

We evaluated the inhibitory effects on Pan02 cells after the treatment with SS LPs (Blank targeting LPs), free TP, TP LPs, and SS-TP LPs at 24 h **(** Figure 4 **)**. Compared to free TP and TP LPs, SS-TP LPs demonstrated the strongest inhibitory effects on Pan02 cell proliferation at 20 nM–640 nM, whereas SS LPs showed no significant inhibitory effect on Pan02 cells at any concentration **(** Figure 4A **)**. The IC_50_ value of SS-TP LPs (43.94 nmol/L ± 1.85 nmol/L) was much lower than those of free TP (70.81 nmol/L ± 4.5 nmol/L) and TP LPs (63.67 nmol/L ± 2.55 nmol/L) in the same TP concentration **(** Figure 4B **)**. Moreover, TP LPs exhibited more effect in cancer cell death compared to free TP, indicating the regular TP liposomes had a very effective drug delivery capacity for free TP. The *in vitro* cytotoxicity data showed that SS-TP LPs had greatly improved anti-tumor activity on Pan02 cells in comparison with free TP and TP LPs, which might be accounted for its enhanced accumulation in Pan02 cells driven by the targeting head SS group and the gradual release of TP from the liposomes.

The apoptosis-inducing effect of SS-TP LPs on Pan02 cells was evaluated to investigate the mechanism that triggers cell death by a flow cytometric analysis. After the incubation with culture medium, free TP, TP LPs, and SS-TP LPs in Pan02 cells for 24 h, the early apoptosis rates were 0, 7.77%, 10.98%, and 15.23% and the late stage apoptosis rates were 0, 8.36%, 13.51%, and 13.19%, respectively (Figure 4C). Undeniably, it was evident that the early apoptosis rate in the SS-TP LPs group was significantly higher than those in the free TP group and TP LPs group. We hypothesized that SS-TP LPs with positive surface charge would target mitochondria specifically and induce early apoptosis, leading to cell death in the Pan02 cells.

The high level of intracellular ROS is more harmful to cancer cells than normal cells, which potentially induces cell necrosis or apoptosis by destroying the mitochondrial membrane (Cheng et al., 2020). Thus, we subsequently investigated whether triptolide improves ROS production in pancreatic cancer cells. The ROS level was determined using the green fluorescent DCFH-DA by fluorescence confocal laser scanning microscopy. As shown in Figures 4D and E, significant increases in the fluorescent signals were observed in the treated Pan02 cells. And the SS-TP LPs group showed the strongest fluorescence intensity of DCFHDA in comparison with the control group and other treated groups (*p* < 0.01). Combined with the previous experimental results, those data indicated that triptolide would trigger the ROS-dependent activation of apoptotic cell death in pancreatic cancer cells.

Here, the possible apoptosis pathway was further validated using the Western blot method. As shown in Figure 4F, the triptolide treatment inhibited considerable anti-apoptosis protein Bcl-2 and pro-caspase 3 in the Pan02 cells. The triptolide promoted the expression of cytochrome C, Bax, and cleaved caspase 3, and the expression levels were further strengthened by SS-TP LPs (Figure 4G). Hence, SS-TP LPs largely improved ROS production, which triggered mitochondria-mediated apoptosis, resulting in the inhibition of BCl-2 and pro-caspase 3, expression of Bax, release of cytochrome C, and activation of caspase 3, leading to apoptosis.

### 3.3 Cellular uptake and mitochondrial outer membrane permeabilization

**FIGURE 5:**
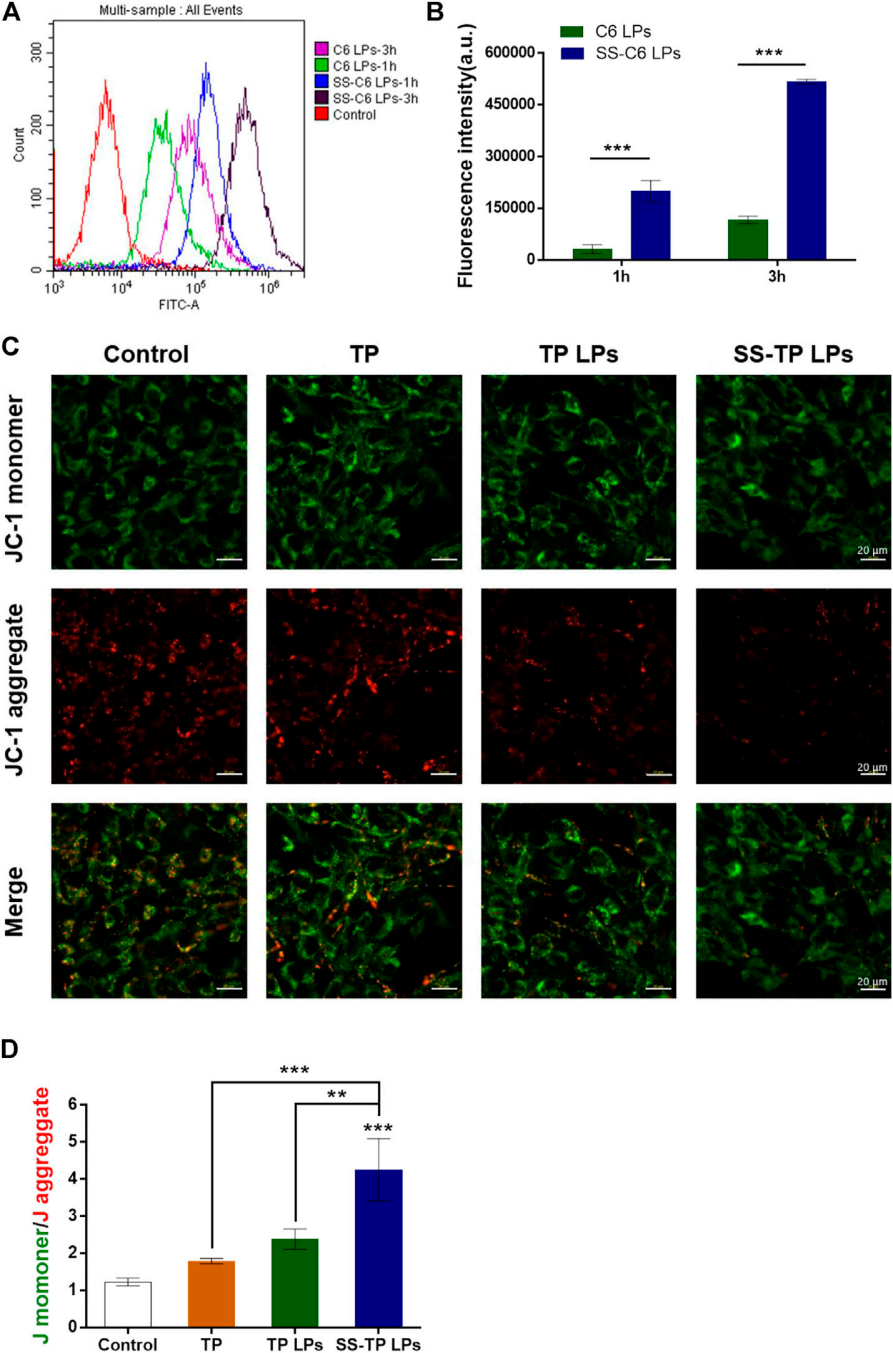
**(A)** Cellular uptake and mitochondrial membrane permeabilization after the treatment with various formulations. **(B)** Uptake of drugs by Pan02 cells at time points of 1 h and 3 h detected by using flow cytometry. **(C)** Comparison of mitochondrial membrane potentials (JC-1 staining) by CLSM images (scale bar = 20 μm). **(D)** Quantification analysis of the ratio of JC-1 monomer (green)/aggregate (red). The results were representative of one of three independent experiments. **p < 0.01 and ***p < 0.001 were considered statistically significant.

Although SS-TP LPs can effectively induce ROS production and trigger mitochondria-mediated apoptosis in Pan02 cells, the mechanism of mitochondrial accumulation of SS-TP LPs is not very well understood yet. To address this challenge, we investigated the cellular uptake mechanism of various coumarin nanoparticles (green) detected by flow cytometry and confocal microscopy. As shown in Figures 5A and B, the fluorescence intensity represents the uptake of coumarin by Pan02 cells. The SS-C6 LPs groups showed over 6-fold fluorescence intensity in Pan02 cells than the C6 LPs groups at 1 h and more than 4-fold at 3 h, indicating a more efficient cellular uptake of SS-TP LPs with a positive charge. The results provided evidence in support of a role for SS-TP LPs in the higher cytotoxicity and apoptosis rate, as discussed previously.

In order to investigate the effects of SS-C6 LPs on mitochondrial outer membrane permeabilization, the mitochondrial membrane potential (Δ_ψm_) was measured using the JC-1 dye by confocal microscopy. The JC-1 dyes potentially accumulate and form aggregates in the normal mitochondria with red fluorescent emission (∼590 nm), whereas the JC-1 dyes show the formation of green fluorescent monomers (∼529 nm) in the depolarized mitochondria. The increasing ratio of green/red fluorescent intensity can be acted as an indicator of mitochondrial depolarization or Δψm (Pathak et al., 2014). After incubation with the free TP, TP LPs, and SS-TP LPs for 24 h, followed by staining with JC-1, the Pan02 cells were imaged by confocal microscopy, respectively. Compared with the control group, the green fluorescence intensity gradually increased, and the red fluorescence intensity decreased in turn from the free TP group, to the TP LPs group, to the SS-TP LPs group, indicating that SS-TP LPs could cause the greatest reduction of the mitochondrial membrane potential (Figure 5C). A quantificational analysis in green/red ratio exhibited a significant increase in SS-TP LPs-treated cells (4.25 ± 0.85), in contrast with the control cells (1.23 ± 0.01), and TP-treated cells (1.79 ± 0.07) and TP LPs-treated cells (2.38 ± 0.27) (Figure 5D). The JC-1 assay revealed that SS-TP LPs significantly induced mitochondrial depolarization (2-fold, maximum) in comparison with TP LPs and free TP. Mitochondrial depolarization leads to permeabilization of the mitochondrial outer membrane, followed by the release of cytochrome C from intermembrane space (Tait and Green, 2010).

### 3.4 Mitochondria localization

**FIGURE 6:**
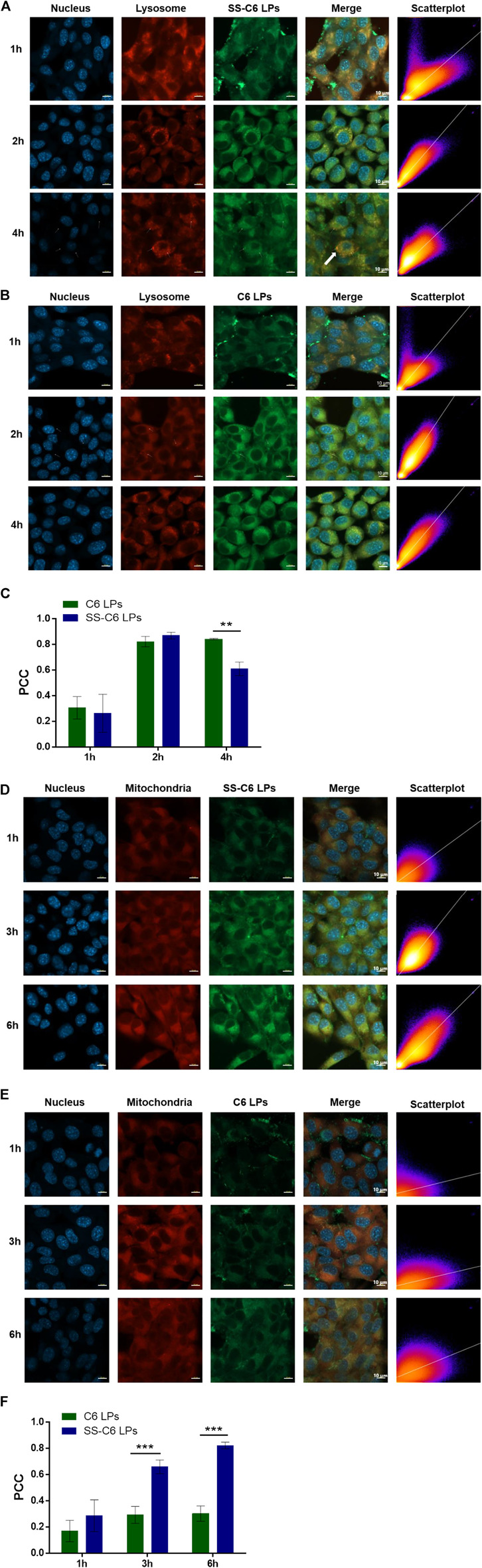
(Continued).

In order to further investigate the actual mechanism of mitochondria homing of the SS-TP LPs, we stained the cell nucleus, lysosomes, and mitochondria with DAPI (blue), LysoTracker^TM^ dye (red), and MitoTracker^TM^ dye (red), respectively. The bright yellow color in the confocal fluorescence images was merged with green and red fluorescence, indicating the SS-C6 LPs (Figure 6A) and C6 LPs (Figure 6B) localized into the lysosomes. As shown in Figures 6A and B, the SS-C6 LPs and C6 LPs rapidly internalized and localized into lysosomes in 2 h. Recent studies show that the size of the lysosomal membrane pores controls the translocation of lysosomal components (Wang et al., 2018a). Cationic amphiphilic drugs or cationic nanoparticles induce lysosomal enlargement (including the pore sizes) and then lysosomal permeabilization, followed by escape from lysosomes (Mallick et al., 2016; Wang et al., 2018b). The quantification analysis using Pearson’s correlation coefficient (PCC) showed that the co-localization volume of C6 LPs in the lysosomes increased from 30.67% to 82.33% to 84.33% when going from 1 to 3 to 4 h, respectively (Figure 6C). However, the co-localization volume of SS-C6 LPs exhibited an initial increase from 26.33% to 87% within 2 h, followed by a decrease to 61% at 4 h, which clearly corroborated that SS-C6 LPs had escaped from the lysosomes.

After lysosomal escape, the SS-C6 LPs might deliver their payloads into mitochondria. To evaluate the mitochondria localization, the SS-C6 LPs and C6 LPs were incubated in Pan02 cells for 1, 3, and 6 h, and then the cells were visualized by confocal microscopy. The bright yellow color, composed of red (MitoTracker dye) and green (coumarin) fluorescence, indicated the co-localization of these two nanoparticles in the mitochondria. Figures 6C and D illustrate that SS-C6 LPs were selectively accumulated into the mitochondria of Pan02 cells in a time-dependent manner. However, the bright yellow fluorescence was not clearly observed after the addition of C6 LPs (Figure 6D). The PCC-based quantification of the co-localization volume of SS-C6 LPs gradually increased from 28.67% to 66% to 82.33% when going from 1 to 3 to 6 h, respectively, indicating that SS-C6 LPs quickly localized into the mitochondria over 6 h (Figure 6E and F), whereas the C6 LPs showed 17%, 29.33%, and 30.33% co-localization regions at 1, 3, and 6 h, which confirmed that SS-C6 LPs has a stronger ability to target mitochondria rather than C6 LPs. The high intracellular and mitochondrial uptake of SS-C6 LPs is consistent with the high cytotoxicity and apoptosis rate, as discussed previously.

### 3.5 The biodistribution of SS-TP LPs in tumor-bearing mice

**FIGURE 7:**
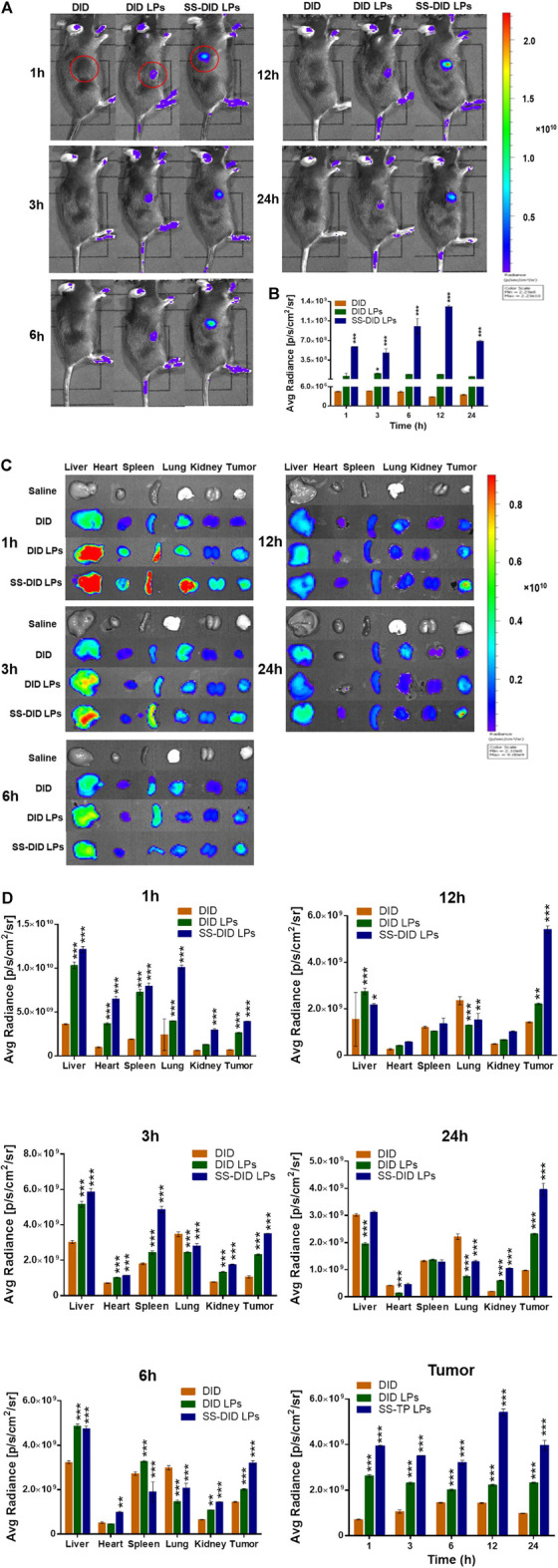
(Continued).

The biodistribution behaviors of SS-TP LPs were investigated in the Pan02-bearing mice to explore the tumor targetability of SS-TP LPs (Figure 7). The fluorescence imaging was performed in the Pan02-bearing mice at 1, 3, 6, 12, and 24 h after tail intravenous injection of saline, DID, DID LPs, and SS-DID LPs, respectively. As shown in Figures 7A and B, the fluorescence was mainly displayed in the tumor region, where the fluorescence intensity of SS-DID LPs was much higher than those of DID and DID LPs at all given time points. Owing to the weak penetration of fluorescence to the thick hair and muscle of mice, the fluorescence could only be displayed on superficial sites such as tumors, ears, and feet. Therefore, we sacrificed the mice at the given time points and performed *ex vivo* imaging of the organs and tumors.

As shown in Figures 7C and D, the saline group showed no fluorescence accumulations in all organs and tumors, and the free DID exhibited very weak fluorescence intensity in the liver, spleen, and lung at all given time points, whereas the DID LPs and SS-DID LPs were mainly accumulated in the liver, spleen, and lung at 1 h post-injection, and then gradually eliminated until 12 h. Conversely, the DID LPs and SS-DID LPs showed increasingly accumulations in the tumor region and peaked at 12 h. In addition, SS-DID LPs showed the highest accumulations in the tumor region within 24 h compared with the other groups. The high accumulation of SS-DID LPs in tumors might be because of improving the systemic circulation of the drug by PEG and the electrostatic attraction between the positive charged stachydrine ester and the negative charged tumor membrane. The results could support the potential application of SS-DID LPs as an effective mitochondria-targeted delivery system for chemotherapeutics.

### 3.6 Antitumor effect of SS-TP LPs in tumor-bearing mice

**FIGURE 8:**
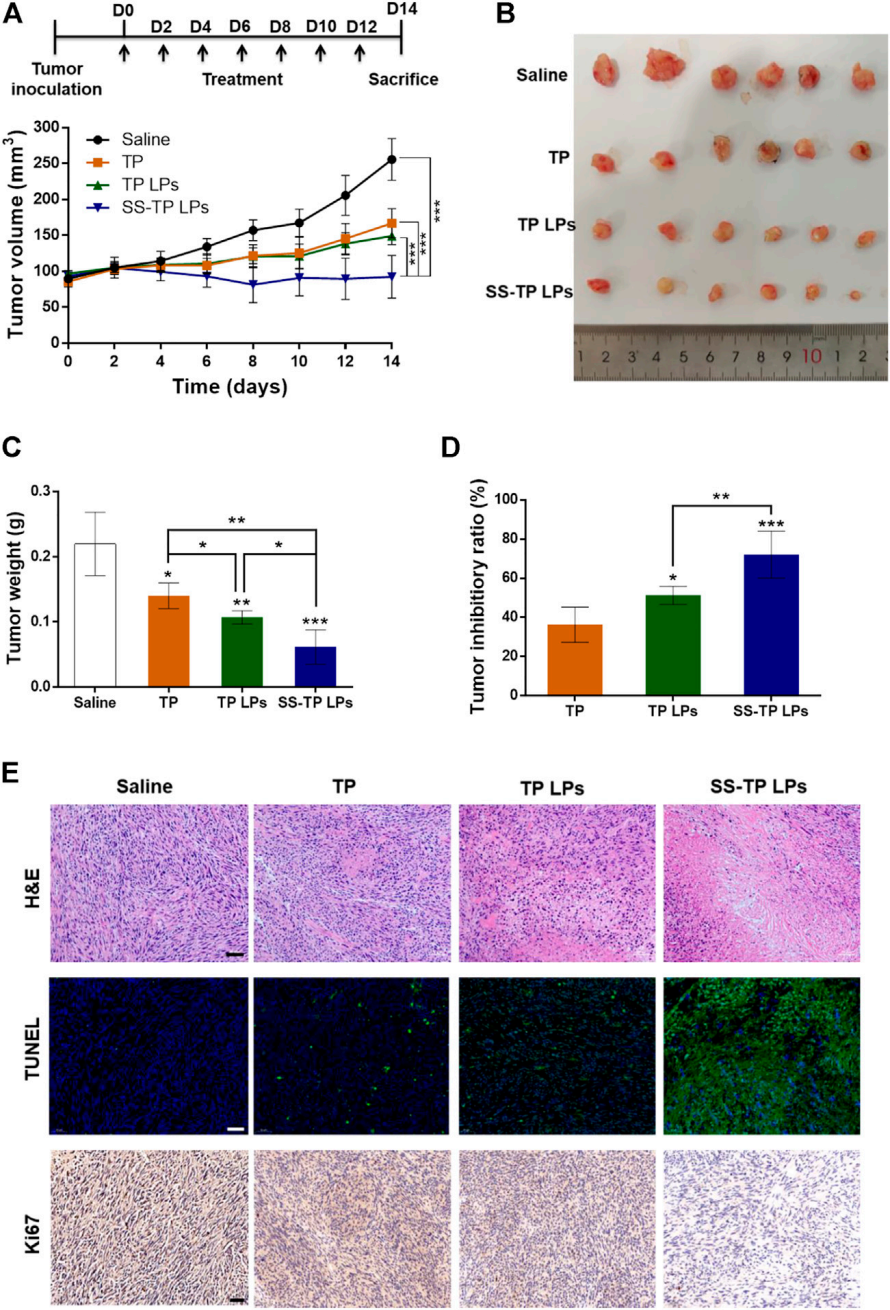
*In vivo* antitumor efficacy of SS-TP LPs in tumor-bearing mice (*n* = 6). **(A)** Tumor growth was measured over time. Treatment drugs were injected every 2 days for a total of seven times. **(B)** Photographs of solid tumors in each treatment group after 14 days. **(C)** Tumor weight variations of different treatment groups at the end of the experiment. **(D)** Tumor growth inhibitory ratios were evaluated from the tumor weight. Data are displayed as mean ± SD. *p < 0.05, **p < 0.01, ***p < 0.001 vs*.* saline. **(E)** HE-stained images of the tumor sections from mice after various treatments (upper); Fluorescence images of tumor slides stained with the TUNEL assay kit after various treatments (middle). The green indicates the cell apoptosis, and the blue indicates nuclei stained with DAPI; Immunohistochemical images of tumor slides stained with the Ki-67 assay kit after various treatments (lower). The brown indicates Ki-67-positive cells. Scale bar = 50 µm.

The tumor inhibitory effect of SS-TP LPs on Pan02 tumor-bearing mice was evaluated by monitoring tumor growth and other pathophysiological changes. As shown in Figures 8A–C, the tumor size and tumor weight were significantly reduced by SS-TP LPs compared with the saline control and TP- and TP LPs-treated groups. SS-TP LPs exhibited the highest tumor inhibitory ratio of 72.06% on day 14 in comparison with the free TP (36.25%, *P* < 0.05) and TP LPs (51.27%, *P* < 0.01) (Figure 8D). This result was consistent with the changes in tumor weight, indicating that the antitumor efficiency of SS-TP LPs was much more excellent than those of TP and TP LPs.

Histological examination of tumor sections revealed significant cell morphology damage and pro-apoptosis and anti-proliferation effects in tumor tissues after treatment of SS-TP LPs (Figure 8E). In the H&E assay, the saline control group showed dense tumor cells with large nuclei, obvious nuclear division and negligible apoptosis levels (Figure 8E, upper). The TP groups and TP LPs groups displayed a few tumor cell necrosis and obvious tumor area shrinkage. Interestingly, the SS-TP LPs group demonstrated dramatic tumor area shrinkage and more void spaces in the core of tumor tissues among all treatment groups. The TUNEL assay showed extensive TUNEL positive staining in the SS-TP LPs group, fewer green positive staining in the TP LPs group and TP group, and negligible positive staining in the control group (Figure 8E, middle). The results confirmed that SS-TP LPs could penetrate deep into the tumor tissues and induce tumor cell apoptosis. IHC staining for the proliferation marker Ki-67 illustrated that SS-TP LPs significantly reduced the number of proliferative cells in comparison with the saline, TP and TP LPs (Figure 8E, lower). The results indicated that the extraordinary anti-tumor efficacy of SS-TP LPs was attributed to its tumor targetability, controlled release of drugs, enhanced apoptosis-induction, and anti-proliferation effect.

### 3.7 *In vivo* safety of SS-TP LPs

**FIGURE 9:**
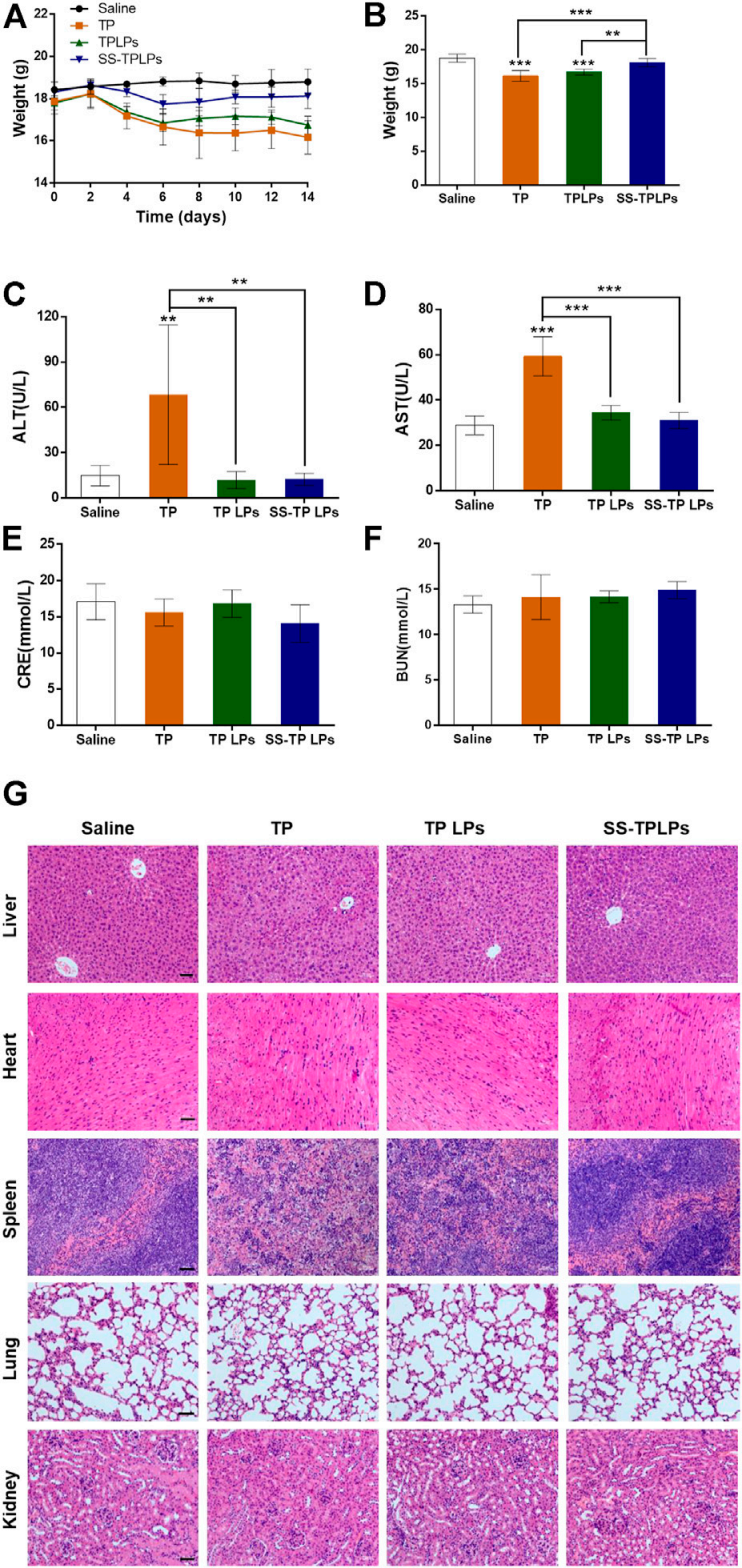
**(A)** Body weight variations of the tumor-bearing mice receiving the indicated treatments. **(B)** Body weights of different treatment groups at the end of the experiment. The hepatic and renal functions were detected by serum alanine aminotransferase **(C)**, aspartate aminotransferase **(D)**, creatinine **(E)**, and blood urea nitrogen **(F)**. **(G)** HE-stained organs isolated from mice after different treatments. Data are represented as mean ± SD; *n* = 6, *p < 0.05, **p < 0.01, ***p < 0.001 vs*.* saline. Scale bar = 50 µm.

Triptolide is a superiorly effective chemotherapeutic drug against pancreatic cancer cells (Chugh et al., 2012; Li et al., 2016; Zhang et al., 2020). But its toxicity is among the critical obstacles to clinical translation. In the current study, the *in vivo* safety of SS-TP LPs in the tumor-bearing mice was evaluated by monitoring body weight change, H&E staining, acute nephrotoxicity, and hepatotoxicity. As shown in Figures 9A and B, compared with the saline groups, the weight changes were not significant in the SS-TP LPs group (*p* > 0.05), while the TP and TP LPs groups exhibited significant weight loss, indicating that SS-TP LPs represent a safe formulation due to its tumor-targeting and the controlled release of drugs. Serum analysis showed that only free TP induced the increase of both AST and ALT, indicating the occurrence of liver injury (Figures 9C–F). However, compared to the control group, both TP LPs and SS-TP LPs groups showed almost no effect on the levels of AST, ALT, BUN, and Crea (*p* > 0.05), which suggested that SS-TP LPs do not induce systemic adverse effects in mice.

The pathological histology analysis showed that no obvious cellular damage induced by SS-TP LPs was observed in the major organs (e.g., liver, heart, spleen, lungs, and kidneys, Figure 9G). However, compared with the control group, the livers in the TP-treated group were hyperemic, mottled, and fragile; the nuclei of hepatic cell emerged pyknosis, rupture, and were even gone; the cytoplasmic staining was heterogeneous with slight cell damage (Figure 9G). In addition, the free TP induced spleen edema and dramatic increase of spleen red pulp, and the morphological structures of the spleen were blurry (Figure 9G). In contrast, the livers and spleens in the TP LPs -treated groups maintained a relatively intact morphological structure, and the cells were almost arranged in an orderly way, but portions of the cellular structures were not very clear. In the control, TP LPs, and SS-TP LPs groups, the renal glomerulus, tubule, and interstitium maintained normal morphological structures (Figure 9G). By comparison, the TP-treated group showed that interstitium was evidently edematous, and lumens emerged narrow or atretic. The boundary of renal tubule epithelial cells was fuzzy. The renal glomerulus was swelling, hyperemic, necrotic, and scattered. Compared to the TP-treated group and TP LPs group, there were no significant histopathological changes in lungs and hearts in the SS-TP LPs group, which showed low toxicity of SS-TP LPs to lungs and hearts (Figure 9G). The results indicated that SS-TP LPs are more tolerable in mice than the TP and TP LPs, which may be attributed to their tumor-targeting and the controlled release of drugs.

## 4 Conclusion

We have synthesized a novel amphiphilic stachydrine derivative (SS) with a positive charge for the mitochondrial targeting in pancreatic cancer cells and modified it onto the triptolide-liposome surfaces. The DPPE-mPEG_2000_ and the suitable particle size of the nanoparticle jointly facilitated the long half-life in blood circulation and the escape from rapid elimination. The SS-TP LPs exhibited more excellent antitumor efficacy with lower adverse effects compared with the free TP and TP LPs. SS-TP LPs were confirmed to be internalized and accumulated into the mitochondria of cancer cells in a time-dependent manner, followed by triggering permeabilization of the mitochondrial outer membrane by inhibiting Bcl-2. The SS-TP LPs subsequently resulted in greater cancer cell death by releasing cytochrome C and initiating a cascade of caspase 3 reactions. The SS-TP LPs were further proven to show specific mitochondria-targeting, excellent therapeutic efficacy, and low toxicity in pancreatic cancer animal models. Our studies suggest that SS-TP LPs can be a promising anticancer drug delivery system for mitochondria-targeted therapy in pancreatic cancer.

## Data Availability

The original contributions presented in the study are included in the article/Supplementary Material; further inquiries can be directed to the corresponding authors.
