# Supplementary material for: A mitochondria-targeted nano-platform for pancreatic cancer therapy
Source: Front Chem. 2022 Sep 21;10:951434. doi: 10.3389/fchem.2022.951434 (PMC9533775; doi:10.3389/fchem.2022.951434)
Supplement: Supplementary file 1 [file DataSheet1.docx]

**A mitochondria-targeted nano-platforms for pancreatic cancer therapy**

**Xiaoke Tan^1,2,#^, Xin Zhu^2,#^, Duanjie Xu^1,2^, Yanmei Shi^1^, Zhenzhen Wang^1^, Mingzhuo Cao^1^, Kai Hu^1^, Lingzhou Zhao^3^, Junwei Zhao^4^, Mingsan Miao ^1,^*, Huahui Zeng ^1,^* , Xiangxiang Wu^1,^***

^1^Academy of Chinese Medicine Sciences, Henan University of Chinese Medicine, Zhengzhou 450046, China.

^2^Pharmacy College, Henan University of Chinese Medicine, Zhengzhou 450046, China.

^3^Department of Nuclear Medicine, Shanghai General Hospital, Shanghai Jiao Tong University School of Medicine, Shanghai 200080, China.

^4^Department of Clinical Laboratory, The First Affiliated Hospital of Zhengzhou University, Zhengzhou 450052, Henan, China.

The characterization spectra of NMR and mass of SS conjugate:

^1^H NMR (500 MHz, Chloroform-*d*) δ 5.36 (t, *J* = 9.7 Hz, 1H), 4.54 (q, *J* = 10.3 Hz, 1H), 4.27 – 4.11 (m, 2H), 4.04 (dd, *J* = 11.6, 8.0 Hz, 1H), 3.75 (s, 3H), 3.22 (s, 3H), 2.76 (dd, *J* = 13.7, 9.7, 4.2 Hz, 1H), 2.42 (d, *J* = 16.8 Hz, 1H), 2.30 – 2.06 (m, 2H), 1.64 (p, *J* = 6.9 Hz, 2H), 1.24 (s, 30H), 0.86 (t, *J* = 6.7 Hz, 3H).

Chemical Formula: C_25_H_50_NO_2_
MS (ESI) m/z, Calcd for C_25_H_50_NO_2_ (M +H) ^+^397.39, found 397.63 (M+H)^+^:
